# From rags to riches: Lactate ascension as a pivotal metabolite in neuroenergetics

**DOI:** 10.3389/fnins.2023.1145358

**Published:** 2023-03-02

**Authors:** Avital Schurr

**Affiliations:** Department of Anesthesiology and Perioperative Medicine, University of Louisville, Louisville, KY, United States

**Keywords:** glucose, glycolysis, lactate, lactate dehydrogenase, mitochondria, NADH (NAD^+^), oxydative phosphorylation (oxphos), trycarboxylic acid (TCA) cycle

## Introduction

Our understanding of neuroenergetics was drastically changed 35 years ago with the revelation that lactate, the end-product of the glycolytic pathway, can be used as a substrate of the mitochondrial oxidative phosphrylation pathway (oxphos) in the absence of glucose (Schurr et al., 1988a). Expectedly, when new scientific discoveries question a long-standing dogma, the idea that lactate is a substrate of oxphos was received with great skepticism from the majority of the scientific community in the field of cerebral energy metabolism (for details see Schurr, 2014). Unfortunately, this skepticism has led many to ignore accumulating data supporting the role of lactate as a substrate of oxphos, which is evidenced in the majority of biochemistry textbook chapters and online biochemistry classes, where pyruvate is still considered to be the end-product of aerobic glycolysis and the oxidative substrate of oxphos. Studies that indicate lactate to be the end-product of glycolysis, either in the absence or presence of oxygen, are generally ignored. Even studies that specifically investigate the role of lactate in neuroenergetics, including several review papers, tend to overlook earlier publications on the topic. Beyond the expected courtesy of citing earlier, relevant work, the omission of such citations in more recent publications contributes to unnecessary delay in advancing the knowledge and understanding of neuroenergetics, the most important biochemical process within the central nervous system (CNS). The past decade also saw multiple studies pointing at additional roles lactate may play in the CNS both as a receptor ligand and a signaling factor. However, those potential roles are a topic to be covered separately. This commentary will relate only to the role of lactate in neuroenergetics. Instead of a repeat attempt at explaining the persisting skepticism about lactate and its role in cerebral energy metabolism (Schurr, 2014), considered here are the scientific data that have placed lactate at the center of neuroenergetics, the process that assures normal and continuous brain function.

## A very brief review of the history of lactate

**Figure 1:**
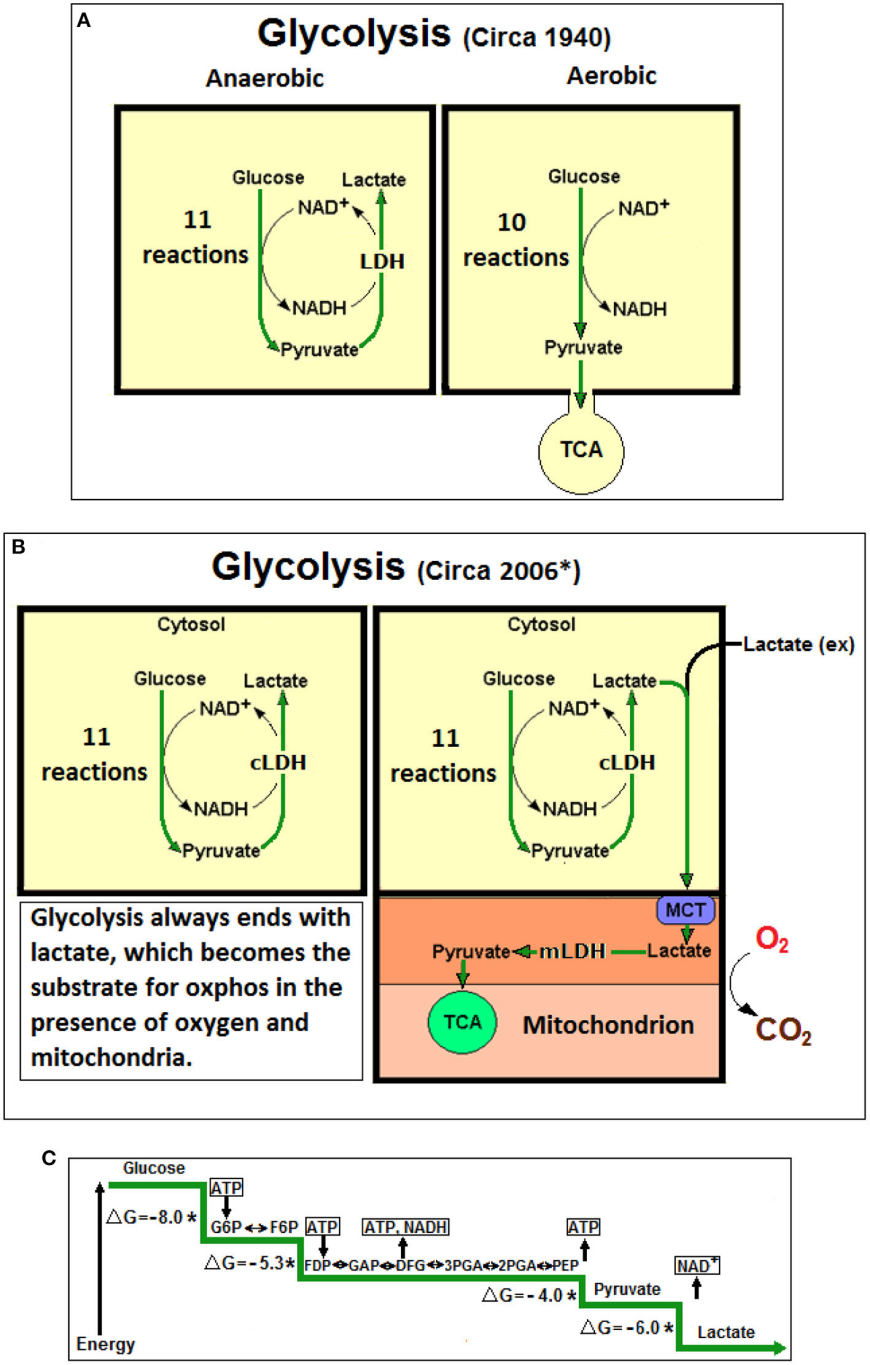
**(A)** At the beginning lactic acid (lactate) was considered to be a waste product of anaerobic glycolysis, not produced during aerobic glycolysis (glycolysis, also known as Embden-Myerhof pathway, was elucidated in 1940). The 11-reaction anaerobic glycolysis, shown on the left, ends with lactate and allows the cyclical production of NAD^+^ from NADH. The 10-reaction aerobic glycolysis, shown on the right, ends with pyruvate, to be used as a substrate of the tricarboxylic acid (TCA) cycle, without any reproduction of NAD^+^ from NADH. **(B)** Over six decades later (*Schurr, 2006), a unified, 11-reaction glycolytic pathway has been suggested where lactate is always its end-product regardless of the presence or absence of oxygen. **(C)** A schematic presentation of the glycolytic free energy change through the eleven enzymatic steps of the pathway. The first step of glycolysis, the phosphorylation of glucose to glucose-6-phosphate by hexokinase, produces the largest free energy change (Δ*G* = −8.0 Kcal), the last step, the conversion of pyruvate to lactate by lactate dehydrogenase (LDH) produces the second largest free energy change (Δ*G* = −6.0 Kcal). No mechanism has been offered in over 80 years to explain how the LDH step could be arrested, as one would expected according to the prevailing aerobic glycolysis dogma, which stands against the strongly favorable thermodynamic conditions. NAD^+^, nicotinamide adenine dinucleotide; NADH, nicotinamide adenine dinucleotide, reduced form; cLDH, cytosolic lactate dehydrogenase (LDH); mLDH, mitochondrial LDH; MCT, monocarboxylate transporter; Lactate (ex), external lactate; ATP, adenosine triphosphate; G6P, glucose 6-phosphate; F6P, fructose 6-phosphate; FDP, fructose 1,6-biphosphate; GAP, glyceraldehyde 3-phosphate; 3PGA, 3-phosphoglycerate; 2PGA, 2-phosphoglycerate; PEP, phosphoenolpyruvate; *Non-equilibrium reaction; Δ*G*, free energy change in Kcal.

In the beginning there was lactic acid in sour milk and from then on this monocarboxylate has been stigmatized, a reputation that remained with lactate when it was shown to be the product of muscular glucose consumption under anaerobic conditions. However, aerobically, muscles can dispose of it (Fletcher and Hopkins, 1907). Similarly, in heart muscle it was observed that under full oxygenation lactate is not produced (Locke and Rosenheim, 1907). Interestingly, in those early days it was assumed that glucose consumption leads to lactate production even in the presence of oxygen and that somehow aerobic conditions allow skeletal and heart muscle to quickly dispose of it. Casting lactate as an undesirable product of energy production *via* glucose metabolism continued unabated throughout the majority of the 20th century. Even a spate of research papers, published in the mid 1920s to early 1930s, on the ability of brain tissue *in vitro* to reduce its lactate levels by supplying it with oxygen, was still interpreted as a disposal mechanism (Holmes and Holmes, 1925a,b; Holmes and Ashford, 1930; Ashford and Holmes, 1931). Consequently, lactate continued to be marked as useless and at times the poisonous end-product of anaerobic glycolysis. This concept entails that lactate is not being produced during aerobic glycolysis, a postulate that led investigators to mark pyruvate as the end-product of aerobic glycolysis, and thus, the substrate of the mitochondrial tricarboxylic acid (TCA) cycle (Krebs and Johnson, 1937a,b; Krebs et al., 1938). Obviously, this concept has taken roots despite the fact that drawing glycolysis as a 10-reaction pathway, ending with pyruvate, rather than an 11-reaction one, ending with lactate, is contradictory to the basic thermodynamic rule of free energy change. Accordingly, lactate has been proposed as the oxidative substrate of mitochondrial oxphos (Schurr, 2006) based on cumulative indirect experimental data (Schurr et al., 1997a,b, 1999) that later were supported by direct biochemical and electrophysiological data (Schurr and Payne, 2007; Schurr and Gozal, 2011, 2015; Schurr, 2014, 2018). Additional studies showing that cerebellum mitochondria metabolize lactate have strengthened this concept (Atlante et al., 2007; Passarella et al., 2008). Nevertheless, in the multiple presentations available online today (for example: Intro Bio Lec. Columbia University; Gibbs Free Energy of Glycolysis Flashcards; Quizlet, Glycolysis, Part 2; Azimuth, https://www.wordpress.com; https://www.youtube.com/watch?v=-u-cQ7Onq_A), an explanation is missing of how the last glycolytic step (step 11), the conversion of pyruvate to lactate is arrested during aerobic conditions despite being the glycolytic step with the a large free energy change (Δ*G* = −6.0 Kcal) second only to the hexokinase reaction (Δ*G* = −8.0 Kcal). Moreover, this doctrinaire presentation ignores the fact that the eleventh glycolytic step allows for the replenishing of the NAD^+^ pool that is necessary to maintain the cyclical nature of glycolysis (Schurr, 2018). It is beyond remarkable that a great majority of biochemists, physiologists, neuroscientists, clinicians and science teachers are still reciting and promoting an incomplete 10-reaction glycolysis that ends with pyruvate, while ignoring the real last glycolytic step (step 11), which must take place under the very thermodynamic rule that applies to all the previous steps. The resistance to abandon the old dogma of glycolysis could be explained by habit of mind (Margolis, 1993; Schurr, 2014). As Figure 1 illustrates, glycolysis ends with lactate, independently of the conditions it proceeds under. Neither oxygen, nor mitochondria halt this outcome and those who claim otherwise have never offered a mechanism that explain how the arrest of the eleventh reaction occurs.

As discussed elsewhere (Schurr, 2014), even four decades after the biochemical sequence of the glycolytic pathway was established, lactate (lactic acidosis) was hypothesized to be the main culprit responsible for the delayed neuronal damage observed after cerebral ischemia (Siesjo, 1981). Ironically, attempting to reproduce enhanced neuronal damage by adding lactic acid to an *in vitro* model of hypoxia/cerebral ischemia had indicated that lowering the pH, either with lactic acid or HCl, protected the tissue against neuronal damage (Schurr et al., 1988b). Simultaneously with the discovery that lactate is a neuronal oxidative substrate (Schurr et al., 1988a). Fox et al. (1988) claimed that glucose consumption during focal physiologic neural activity is non-oxidative. The description “non-oxidative glucose consumption” is also known as the “Warburg Effect” (Warburg et al., 1927) and “aerobic glycolysis.” Non-oxidative glucose consumption and aerobic glycolysis are terms born due to the decision to describe the glycolytic pathway as having two separate outcomes, depending on the presence or absence of oxygen namely, “aerobic” and “anaerobic” glycolysis. Accordingly, the former ends with pyruvate and the latter with lactate. In 1940 our knowledge regarding the role of mitochondria was non-existent and any hint of oxphos participation in energy metabolism was limited to the fact that phosphate participates in the metabolic reactions. The idea that the presence of oxygen somehow determines the fate of glycolytic glucose metabolism became the tenet, which still occupies both textbooks and minds.

## Just glycolysis: Devoid of the misleading aerobic and anaerobic prefixes

Considering the above mentioned history, any observation of glucose metabolism not accompanied by oxygen consumption is categorized as aerobic glycolysis. Of course, red blood cells, devoid of mitochondria, but rich in oxygen, produce ATP by “aerobic” glycolysis. Proliferating cancer cells do it (Warburg et al., 1927), so does the Na/K-ATPase pump (Balaban and Bader, 1984) and, since 1988, focal neural cells during physiologic activity do it too (Fox et al., 1988). Separating glycolysis into aerobic and anaerobic pathways greatly confuses both the basic understanding of its metabolic role in the CNS and most likely in every other tissue as well as its diagnostic and medical applications. With the development and advancement of our knowledge and understanding of mitochondria and their role in respiration and ATP production, the designation of aerobic and anaerobic glycolysis was changed such that the former requires not only the presence of oxygen, but also the presence of mitochondria, while the latter requires the absence of both (Naifeh et al., 2022). Nevertheless, a mechanism by which the eleventh step of glycolysis namely, the enzymatic conversion of pyruvate to lactate by lactate dehydrogenase (LDH), is somehow “turned off” when oxygen and mitochondria are present, has never been offered or pursued, let alone resolved. This “mystery” continues to accompany every online presentation of glycolysis, in published papers and in textbooks. As indicated above, a habit of mind (Margolis, 1993; Schurr, 2014) must be responsible for the continuous promotion of a concept that is fundamentally off the mark. That the presence of oxygen has nothing to do with glycolysis ending with the tenth reaction and somehow arresting the eleventh (LDH) one from proceeding, is absolutely obvious. Similarly, no mechanism has been offered yet, to explain how the presence of mitochondria switch the glycolytic pathway from 11 to 10 reactions. Glycolysis is a pathway present in every other bodily tissue beside the CNS and multiple studies over the past several decades exhibited mitochondrial utilization of lactate as a substrate of oxphos, while other exhibited the presence of LDH intra-mitochondrially (Brooks, 1985, 1998, 2000, 2002a,b; Larrabee, 1996; Hu and Wilson, 1997; Brooks et al., 1999, 2022; Magistretti et al., 1999; Valenti et al., 2002; Mangia et al., 2003; Pellerin and Magistretti, 2003; de Bari et al., 2004, 2010; Kasischke et al., 2004; Serres et al., 2005; Hashimoto et al., 2006, 2008; Schurr and Payne, 2007; Hashimoto and Brooks, 2008; Passarella et al., 2008; Elustondo et al., 2013; Rogatzki et al., 2015; Hui et al., 2017).

Therefore, it is even more bewildering that the majority of recently published general information, both in print and online, continues to completely ignore the data proving that the glycolytic pathway, regardless of the presence of oxygen and/or mitochondria, always produces lactate as its end-product. The terminology “aerobic” and “anaerobic” glycolysis is meaningless, confusing and should be eliminated (Schurr and Passarella, 2022). Interestingly, as early as 1984 it was suggested to use the term “non-robic” glycolysis instead of the terms “aerobic” and “anaerobic” (Brooks and Fahey, 1984), a suggestion that clearly was not accepted. It is worthwhile mentioning that neuroenergetics is not the only field of research where skepticism about the roles of glycolysis and lactate persists, particularly in muscle energy metabolism and more generally in exercise physiology. Moreover, advances in treatment of neurological conditions, such as traumatic brain injury (TBI), are driving a revision in our understanding of the roles of glycolysis and lactate in these clinical settings.

A new study made use of an ultrasensitive, highly responsive, radiometric lactate sensor that enables the user to monitor subtle lactate fluctuations in living cells and animals. The investigators have definitively demonstrated that lactate is highly enriched in mammalian mitochondria and the important role it plays in cellular metabolism (Li et al., 2022). Unfortunately, the authors chose not to cite a great number of relevant studies that support their findings and could greatly strengthen their important discoveries. Nevertheless, the reported findings confirm the assertion that lactate is the glycolytic end-product and the mitochondrial substrate for the tricarboxylic acid cycle (TCA). The investigators showed that the lactate pool intra-mitochondrially in glucose-fed cell lines was up to10 times higher than that in the cytosol. Moreover, the “*lactate levels are sensitive to changes in glucose levels and metabolism*,” where they increased when glucose levels did and *vice versa*. In addition, this study found that the mitochondrial lactate enrichment was inhibited by the use of the mitochondrial uncoupler, carbonyl cyanide 3-chlorophenylhydrazone (CCCP), indicating that such enrichment is due to oxphos being coupled to the mitochondrial electron transport chain activity.

## Summary

This opinion is another attempt to persuade scientists, clinicians, teachers and students alike to discontinue the use of the archaic and misleading prefixes “aerobic” and “anaerobic” always attached to the term “glycolysis.” They should be discontinued and “glycolysis” must stands alone. Since it is now clearly evident that glycolysis is a pathway that consists of eleven enzymatic reactions the last one of which always terminates with the production of lactate and NAD^+^, independent of the presence or absence of oxygen and mitochondria, “aerobic” and “anaerobic” glycolysis cannot be differentiated from one another. This antiquated differentiation was based on the assumption that the glycolytic pathway has two different outcomes/end products, pyruvate under aerobic and lactate under anaerobic conditions. The fact that lactate is the one and only terminal glycolytic product, any prefix assigned to “glycolysis” is, in essence, meaningless. This is especially glaring when the term “aerobic glycolysis” that is in use today means that the pathway ends with lactate as its final product without any oxygen consumption, despite the presence of oxygen (Schurr and Passarella, 2022). Consequently, pyruvate should be considered an intermediate, not different from any other glycolytic intermediate. Lactate produced in the cytosol is shuttled *via* monocarboxylate transporters (MCTs) intracellularly or intercellularly into mitochondria, where it is then converted to pyruvate intra-mitochondrially *via* a mitochondrial LDH (mLDH) to enters the TCA cycle (Figure 1B). In conclusion, the glycolytic pathway's main purpose is the production of two terminal products; lactate, the substrate of the mitochondrial TCA cycle and NAD^+^, a redox cofactor utilized both extra- and intra-mitochondrially.

## Author contributions

The author confirms being the sole contributor of this work and has approved it for publication.
